# Microglial Implication in Parkinson’s Disease: Loss of Beneficial Physiological Roles or Gain of Inflammatory Functions?

**DOI:** 10.3389/fncel.2018.00282

**Published:** 2018-08-30

**Authors:** Cynthia Lecours, Maude Bordeleau, Léo Cantin, Martin Parent, Thérèse Di Paolo, Marie-Ève Tremblay

**Affiliations:** ^1^Axe Neurosciences, Centre de Recherche du CHU de Québec, Université Laval, Quebec, QC, Canada; ^2^Faculté de Pharmacie, Université Laval, Quebec, QC, Canada; ^3^Integrated Program of Neuroscience, Faculty of Medicine, McGill University, Montréal, QC, Canada; ^4^Département de Chirurgie, Faculté de Médecine, Université Laval, Quebec, QC, Canada; ^5^CERVO Brain Research Centre, Quebec, QC, Canada; ^6^Département de Psychiatrie et Neurosciences, Faculté de Médecine, Université Laval, Quebec, QC, Canada; ^7^Département de Médecine Moléculaire, Faculté de Médecine, Université Laval, Quebec, QC, Canada

**Keywords:** aging, chronic stress, inflammation, microglia, phagocytosis, Parkinson’s disease

## Abstract

Microglia, often described as the brain-resident macrophages, play crucial roles in central nervous system development, maintenance, plasticity, and adaptation to the environment. Both aging and chronic stress promote microglial morphological and functional changes, which can lead to the development of brain pathologies including Parkinson’s disease (PD). Indeed, aging, and chronic stress represent main environmental risk factors for PD. In these conditions, microglia are known to undergo different morphological and functional changes. Inflammation is an important component of PD and disequilibrium between pro- and anti-inflammatory microglial functions might constitute a crucial component of PD onset and progression. Cumulated data also suggest that, during PD, microglia might lose beneficial functions and gain detrimental ones, in addition to mediating inflammation. In this mini-review, we aim to summarize the literature discussing the functional and morphological changes that microglia undergo in PD pathophysiology and upon exposure to its two main environmental risk factors, aging, and chronic stress.

## Introduction

Parkinson’s disease (PD) affects one to two individuals per 1000 (Tysnes and Storstein, 2017), making it the most common neurodegenerative movement disorder (Morin et al., 2014). The diagnosis is based on four clinical cardinal signs: rigidity, bradykinesia, resting tremors, and postural instability (Jankovic, 2008). PD is often preceded by a prodromal stage, which includes non-motor symptoms like mood and sleep disorders (Poewe, 2008). In PD, motor symptoms arise from the progressive degeneration of dopaminergic (DA) neurons in the *substantia nigra* (SN) *pars compacta*. DA neurons innervate the striatum and their degeneration is associated with a significant decrease of DA striatal content (Morin et al., 2014; Tysnes and Storstein, 2017). DA neurons loss is often associated with an accumulation of Lewy bodies (LB), which are formed by the aggregation of misfolded α-synuclein, mainly in the SN, but also across several brain regions (Tansey and Goldberg, 2010). PD pathogenesis is associated with genetic variations and environmental risk factors that mainly comprise aging and chronic psychological stress, as well as infection, brain trauma, and exposure to pesticides or herbicides (Tansey and Goldberg, 2010; Schapira and Jenner, 2011; Vyas et al., 2016; Niraula et al., 2017). Levodopa (L-DOPA) is the gold-standard symptomatic treatment for PD, as no DA agonist demonstrates an equal efficacy on motors symptoms. However, adverse effects limit its chronic use. Within 5–10 years of treatment, most patients experience motor complications including L-DOPA-induced dyskinesia (LID), abnormal involuntary movements that can be more debilitating than the disease itself (Mercuri and Bernardi, 2005).

Inflammation, among the central nervous system (CNS) and periphery, is also a main hallmark of PD (Vawter et al., 1996; Nagatsu et al., 2000; Imamura et al., 2003; Mount et al., 2007; Litteljohn and Hayley, 2012; Doorn et al., 2014). In the CNS, microglia which are known as the resident immune cells were proposed to mediate the inflammatory response in PD. The two main environmental risk factors for PD, aging, and chronic stress, are linked to increased levels of pro-inflammatory mediators in the CNS and periphery (Vyas et al., 2016; Niraula et al., 2017; Tian et al., 2017). Nevertheless, the implication of microglia in the development and progression of PD is still unclear, and it remains undetermined whether their alterations are a cause or consequence of DA neurons loss (Le et al., 2016). Microglia were recently shown to exert throughout the lifespan crucial physiological roles (Tay et al., 2018), which could become compromised and contribute to PD pathophysiology. Transcriptomic studies also shed light on the complex signature of microglia, defining several phenotypes across contexts of health and disease (Butovsky et al., 2014; Gosselin et al., 2017; Hanamsagar et al., 2017; Wlodarczyk et al., 2017). In the present mini-review, we aim to summarize microglial functions in health and their potential implications in PD.

## Diverse Roles of Microglia in Health

The origin of microglia has long been a subject of debate until elegantly designed *in vivo* lineage studies in mice identified erythromyeloid cells from the embryonic yolk sac as their progenitors (Ginhoux et al., 2010; Schulz et al., 2012; Kierdorf and Prinz, 2013; Gomez et al., 2015; Hoeffel et al., 2015). These progenitors colonize the brain during the first trimester of fetal development, in both rodents, and humans, then mature into microglia (Kierdorf and Prinz, 2013). Thereafter, microglial pools are maintained by self-renewal, at least under normal physiological conditions (Hashimoto et al., 2013; Askew et al., 2017).

**FIGURE 1:**
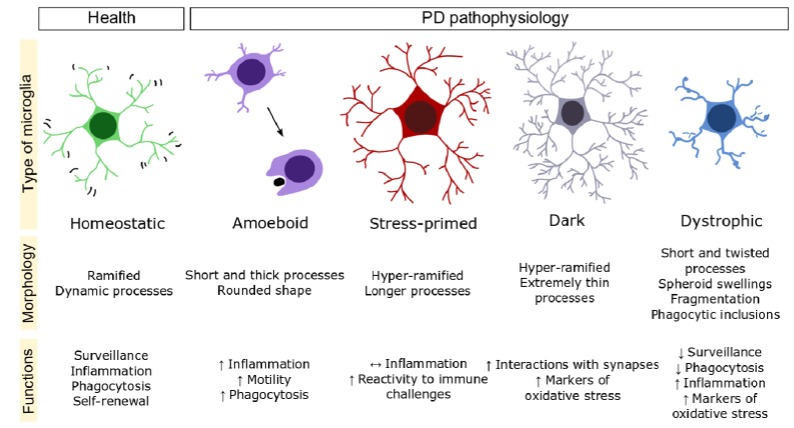
Microglial phenotypes that could be differently implicated in PD. Schematized morphologies are represented and their main characteristics summarized. The following symbols are used; ↑, increased; ↔, unchanged; ↓, decreased.

Mature microglia which display a ramified morphology, referred to as homeostatic microglia, constantly survey the CNS environment and contribute to its maintenance and plasticity through specific molecular pathways (Tremblay et al., 2011; Kierdorf and Prinz, 2013; Nayak et al., 2014; Schafer and Stevens, 2015; Tay et al., 2017). In particular, homeostatic microglia contribute to synaptogenesis, synaptic pruning, and myelination (Schafer and Stevens, 2015; Kaur et al., 2017; Li and Barres, 2017; Paolicelli and Ferretti, 2017; Tay et al., 2017; Low and Ginhoux, 2018). Microglia are also required for the adaptation of the brain and behavior to the living environment (Tremblay et al., 2011; Schafer and Stevens, 2015; Tay et al., 2017, 2018). Upon injury or infection, and even chronic psychological stress, microglia undergo various morphological and functional changes often designated as microglial “activation” or reactivity to pathological or traumatic challenges (Nayak et al., 2014; Tay et al., 2017). Morphological and functional changes of microglia also occur during aging where these cells become “senescent,” i.e., impaired in their surveillance and response to injury (Streit et al., 2014). Considering that changes in microglial density and morphology profoundly impact on their functions (summarized in **Figure 1**), these findings indicate that microglia could play an important role in PD.

## Microglia in Parkinson’s Disease Pathophysiology

In macaque monkeys treated with the neurotoxin 1-methyl-4-phenyl-1,2,3,6-tetrahydropyridine (MPTP), microglia immunopositive (+) for major histocompatibility complex (MHC) class II were found to be highly heterogeneous. They showed ramified, amoeboid or multinucleated morphologies, in the SN, nigrostriatal tract, and globus pallidus. Of note, MHC class II is considered a marker of antigen presentation (Weenink and Gautam, 1997). However, in the striatum of macaque monkeys receiving MPTP, microglia mainly displayed a ramified morphology with little evidence of active phagocytosis associated with the accumulation of fat granules in their processes (Hurley et al., 2003). By contrast, amoeboid microglia were observed in both the SN and striatum of mice exposed to MPTP (Kurkowska-Jastrzebska et al., 1999; Wu et al., 2002). Differences in MPTP administration paradigms or between species could explain this apparent discrepancy (Hurley et al., 2003). In fact, PD progression in humans has been shown to be best recapitulated in non-human primates (**Table 1** summarizes different animal models used in PD research) (Grow et al., 2016).

**Table 1:** Summary of the main animal models used in PD research.

Animal models		Symptoms	
	Motor	Degeneration	LID	Others
**Rodent**				
6-OHDA rat model	↓ Locomotion, modified behavior	↑↑↑ SN loss, ↑↑↑ striatal DA loss, no LB, unilateral lesion	AIM scale available to measure LID	Not specific to DA neurons, not progressive, DA priming needed for antiparkinsonian effects
6-OHDA mouse model	-	-	Poor LID	-
MPTP mouse model	↓ Locomotion, bradykinesia	↑↑↑ SN neuron loss, ↑↑↑ striatal DA neuron loss, no LB	Need high L-DOPA doses to induce AIM, poor LID	-
Rotenone rat model	↓ Locomotion	↑↑ SN neuron loss, ↑↑↑ striatal DA loss, LB	-	-
α-synuclein transgenic mouse model	Modified behavior, ↓ or ↑ motor activity	SN neuron loss (variable), striatal DA loss, LB (old animals)	Poor LID	-
**Non-human primate**				
MPTP cynomolgus and rhesus macaque model	Modified behavior, tremor and rigidity, ↓ locomotion	↑↑↑ SN neuron loss, ↑↑↑ striatal DA loss, no LB (but α-synuclein accumulation), bilateral lesion (if systemic)	LID (best model), LID reappearance after L-DOPA withdrawal, different pattern of LID: stereotypic behaviors	First L-DOPA dose induces antiparkinsonian response, best to test surgical treatments
MPTP marmoset model	Modified behavior, tremor and rigidity, ↓ locomotion, movement indistinguishable, hyperkinesia	↑↑↑ SN neuron loss, ↑↑↑ striatal DA loss, no LB (but α-synuclein accumulation)	LID	Allows to test surgical treatments, not the best to test anti-dyskinetic drugs
MPTP squirrel monkey model	Modified behavior, tremor and rigidity, ↓ locomotion	↑↑↑ SN neuron loss, ↑↑↑ striatal DA loss, possibility of LB (α-synuclein aggregates)	LID, have LID under unlesioned conditions	Limited for study of motor complication (no abnormal PD movements observed)

*The following symbols are used; ↑, increase; ↓, reduction; ↑↑↑, severe; ↑↑, moderate, summary of (Tieu, 2011; Blesa and Przedborski, 2014; Morin et al., 2014; Jagmag et al., 2016; Morissette and Di Paolo, 2018).*

Various brain regions (e.g., pons, basal ganglia, striatum, frontal, and temporal cortices) of PD patients also showed increased binding of the radiotracer ^11^C-(R)-PK11195 compared to age-matched healthy controls by positron emission tomography (Ouchi et al., 2005; Gerhard et al., 2006). The radiotracer ^11^C-(R)-PK11195 binds to 18-kDa translocator protein (TSPO) expressed mainly by microglia, in association with inflammatory stimuli (Mondelli et al., 2017). In the SN of post-mortem PD samples, MHC class II+ microglia were first described in 1988 (McGeer et al., 1988). Since then, other studies confirmed the presence of reactive microglia in the SN of PD patients (Hirsch and Hunot, 2009). Besides MHC class II, microglia were shown to express intracellular adhesion molecule (ICAM)-1, integrin receptors CD11a, the lysosomal activity marker CD68, and the scavenger receptor TLR2 in the SN, putamen, and hippocampus of PD patients (Imamura et al., 2003; Doorn et al., 2014). Microglia also stained positively for pro-inflammatory cytokines such as tumor necrosis factor (TNF)α and interleukin (IL)-6 in the striatum of PD patients (Imamura et al., 2003). Other investigators nevertheless failed to observe evidence of microglial reactivity in the same region of PD patients (Knott et al., 1999; Mirza et al., 2000). Cytokines such as IL-1β, IL-2, IL-4, IL-6, TNFα, transforming growth factor (TGF)α, and TGFβ1 were also increased at the protein level in the striatum, and in the ventricular and lumbar cerebrospinal fluid of PD patients (Vawter et al., 1996; Nagatsu et al., 2000). Additionally, high levels of interferon (IFN)γ were measured in blood plasma from PD patients (Mount et al., 2007). Taken together, this data suggests that PD patients possess an increased brain inflammatory status. Neurotoxic reactive species that microglia can produce, such as superoxide and nitric oxide, were proposed to induce cellular stress and, in turn, contribute to neuronal loss in PD (Block et al., 2007; Le et al., 2016). Moreover, the cerebrospinal fluid of PD patients was shown to be toxic to DA neurons *in vitro* due to the high concentration of cytokines and auto-antibodies against quinone proteins altered by DA oxidation (He et al., 2002; Nagatsu and Sawada, 2005).

### Loss of Beneficial Physiological Functions

Signaling between the microglial complement receptor 3 (CR3) and its ligand, the complement component C3, enriched at synapses, plays a key role in synaptic pruning during brain circuits refinement (Schafer et al., 2012). In rats chronically receiving rotenone, a pesticide acting as a mitochondrial complex I inhibitor, CR3+ microglia were more abundant in the striatum and the SN. They also possessed an enlarged cell body with shorter, stubby processes in these two regions, contrary to the cerebral cortex, suggesting an exacerbated phagocytic activity (Sherer et al., 2003). Microglia release neurotrophic and anti-inflammatory factors that promote neuronal survival (Le et al., 2016). These cells can also modulate the formation of dendritic spines through the release of brain-derived neurotrophic factor (BDNF) in mouse primary motor cortex, a role that was required for motor learning and procedural memory (Parkhurst et al., 2013). In PD, BDNF levels were reduced in the nigrostriatal region and/or cerebrospinal fluid of PD patients and animal models, notably exposed to MPTP or 6-hydroxydopamine (6-OHDA) (Nagatsu and Sawada, 2005). Furthermore, glial-derived neurotrophic factor (GDNF) was shown to protect and rescue DA neurons from degeneration in models, including rats exposed to methyl-4-phenylpyridinium (MPP^+^), the active metabolite of MPTP (Ding et al., 2004; Nam et al., 2015). Additionally, in a mouse model overexpressing human mutant α-synuclein, within neurons mostly of the spinal cord, an increase in ionized calcium binding adaptor molecule 1 (IBA1)+ microglial staining was measured in this region alongside an increased co-expression of AXL (Fourgeaud et al., 2016). With TYRO3 and MER, AXL is part of the TAM receptor family of tyrosine kinases that regulates microglial phagocytic removal of apoptotic cells, notably during adult neurogenesis. In the α-synuclein transgenic mouse, loss of both receptors modestly prolonged the lifespan (Fourgeaud et al., 2016). The authors speculated that microglia might remove distressed motor neurons in PD, through TAM receptor-mediated “phagoptosis” of living neurons causing their death (Brown and Neher, 2012), thus accelerating PD progression (Fourgeaud et al., 2016). In this case, a beneficial microglial function was proposed to become detrimental upon disease.

### Gain of Detrimental Inflammatory Functions

Midbrain DA neurons may be particularly vulnerable to detrimental microglial functions due to the abundance of these cells in this region (Lawson et al., 1990). This susceptibility is also conferred by the enrichment of DA neurons with iron, a redox active element, associated with antioxidant glutathione deficiency and monoamine oxidase activity, which all contribute to DA oxidation resulting in the production of reactive species (Block et al., 2007; Tansey and Goldberg, 2010; Wang and Michaelis, 2010). This susceptibility of the SN was further emphasized by the finding that local lipopolysaccharide (LPS; a bacterial component) injection into the SN, hippocampus or cerebral cortex of wild-type rats induced neuronal loss only in the SN (Kim et al., 2000). Further *in vitro* characterization of neuron-glial cultures identified a key role for microglia in the regional sensitivity to LPS. Indeed, supplementation of microglia into cortical neuron-glial cultures was sufficient to promote LPS-induced neurotoxicity (Kim et al., 2000). Microglia might over-produce pro-inflammatory mediators and reactive species, notably when performing phagocytosis, which could lead to neuronal damage and in turn contribute to sustaining inflammation in PD (Whitton, 2007; Tansey and Goldberg, 2010). In rat primary neuron-glial cell cultures, the exogenous application of α-synuclein aggregates induced microglial transformation into amoeboid cells, which produced reactive species resulting in DA neurons loss (Zhang et al., 2005). *In vitro*, neuromelanin (NM), a dark pigment formed by melanin that is found in catecholaminergic neurons (containing DA or norepinephrine) (Fedorow et al., 2005), induced loss of DA neurons when added to human primary mesencephalic neuron-glia cultures (Zhang et al., 2011). The phagocytic clearance of NM by microglia also induced the production of superoxide, nitric oxide, hydrogen peroxide and pro-inflammatory TNFα and IL-6, which could be prevented by genetic deletion of CR3 *in vitro* (Zhang et al., 2011). Besides NM, matrix metalloproteinase 3 (MMP3) and α-synuclein, which are released by degenerating DA neurons, promote microglial production of reactive species (Block et al., 2007).

## Microglia in Aging and Chronic Stress

### Normal Aging and Microglial Alterations

Aging is associated with an increased expression level of pro-inflammatory cytokines (e.g., IL-1β, IL-6, and TNFα) as well as decreased expression level of anti-inflammatory cytokines (e.g., IL-10) and anti-oxidants (e.g., glutathione levels) in rodent brain (Frank et al., 2006; Sierra et al., 2007; Ritzel et al., 2015). Furthermore, during aging, LPS-immune-challenged mice display an exacerbated inflammatory response (Godbout et al., 2005; Sierra et al., 2007; Njie et al., 2012). With relevance to PD, analyses of IBA1+ microglia in the SN and striatum of wild-type mice, from birth until 24 months of age, revealed that cellular density is decreased while clustering is increased after 18 months of age in both regions (Sharaf et al., 2013). Moreover, microglia underwent dystrophic morphological changes in the SN (see **Figure 1**), such as reduced ramifications, starting at 12 months (Sharaf et al., 2013). In aged rhesus monkeys, an increase in human leukocyte antigen-DR (HLA-DR), a component of MHC class II+ microglia, and similar morphological alterations were observed in the SN, together with an increased prevalence of amoeboid-shaped microglia, upon administration of the MPTP toxin (Kanaan et al., 2008).

### Chronic Stress and Pathological Aging

Aging can induce important alterations of brain homeostasis notably through its effects on microglia. In addition, aging might become “pathological” under the influence of an environmental risk factor, such as chronic psychological stress, thus triggering disease onset and progression (Streit et al., 2014).

Chronic psychological stress can accelerate cellular aging by acting on both oxidative stress and inflammation (Tay et al., 2017; Tian et al., 2017). Upon stress, microglia become “primed” and show exaggerated response to a subsequent challenge (Cunningham et al., 2005; Frank et al., 2007). Other than the neuroinflammation changes, chronic restraint stress in otherwise healthy rodents was associated with a loss of neurons expressing tyrosine hydroxylase (TH), the enzyme that converts L-DOPA into DA, in the SN (Sugama et al., 2016; Ong et al., 2017). This TH+ neuronal loss correlated with an increase of insoluble α-synuclein monomers leading to the formation of aggregates and decreased numbers of IBA1+ microglia in the SN (Ong et al., 2017). Furthermore, when MPTP was administered after inducing stress, using the same paradigm, the loss of TH+ neurons in the SN was found to be more important in stressed rats than in unstressed littermates, which also displayed motor learning deficits (assessed with the rotarod) (Lauretti et al., 2016). However, different outcomes of stress on DA neurons were reported according to the type of stressor used and the brain region examined (Belujon and Grace, 2015). The overall evidence nevertheless suggests a close relationship between chronic stress and PD, which highlights the importance of investigating microglial changes as a contributing factor to PD pathophysiology.

Another subset of microglia, “dark microglia” identified by electron microscopy, was observed in adult mice exposed to maternal immune activation (Hui et al., 2018), chronic unpredictable stress, or aging (Bisht et al., 2016). Dark microglia display several markers of oxidative stress including a condensed cytoplasm and nucleoplasm, which led to their name, accompanied by dilation of the endoplasmic reticulum and Golgi apparatus, as well as mitochondrial alteration. They are highly ramified (see **Figure 1**) with their processes extensively encircling excitatory synapses and making direct contacts with synaptic clefts (Bisht et al., 2016) suggesting an involvement in synaptic remodeling under pathological or traumatic conditions. The involvement of dark microglia in PD still remains unknown, however.

## Conclusion

Overall, various studies using *in vivo* and *in vitro* approaches have improved our knowledge of microglial involvement in PD despite the differences in paradigms and species used. Aging and chronic stress, two main environmental risk factors for PD, exacerbated inflammation and altered microglial functions. These changes might trigger pathological pathways notably in vulnerable CNS regions, such as the SN. Microglia produce both pro- and anti-inflammatory mediators, but upon neurodegeneration this tight equilibrium might get disrupted and become mainly pro-inflammatory. The death of DA neurons and associated reactive species production lead to pro-inflammatory and “phagoptotic” microglial phenotypes. Considering the inflammatory component of PD, it is important to study microglial implication with its onset, progression, and symptomatic treatment using L-DOPA for a translation to human patients. A better comprehension of the roles of different microglial phenotypes in PD might one day help to find anti-parkinsonian and/or anti-dyskinetic drugs targeting microglia.

## Author Contributions

CL, MB, and M-ÈT designed the review outline and wrote a first version of the manuscript. MP, LC, and TD revised the manuscript and contributed to the subsequent versions.

## Conflict of Interest Statement

The authors declare that the research was conducted in the absence of any commercial or financial relationships that could be construed as a potential conflict of interest.
